# Robotic assisted two-level prone lateral lumbar interbody fusion

**DOI:** 10.3171/2026.4.FOCVID2632

**Published:** 2026-07-01

**Authors:** Abdullah Al Tekreeti, Heegook Yeo, Seamus Bartlett, Jared Reese, Anisse N. Chaker, Victor Chang

**Affiliations:** 1 Wayne State University School of Medicine, Detroit; and; 2 Department of Neurosurgery, Henry Ford Health, Detroit, Michigan

**Keywords:** prone LLIF, single-position lumbar fusion, intraoperative navigation, robotic assisted surgery, coronal deformity

## Abstract

Prone LLIF is a single-position technique enabling circumferential lumbar fusion without repositioning, potentially improving efficiency and safety. Early data show technical success (approximately 89%–97%) and low vascular or visceral injury rates, with mostly transient hip flexor weakness or sensory symptoms. The authors present the case of a 68-year-old female with L3–4 and L4–5 laterolisthesis, coronal curvature, and severe disc collapse causing foraminal stenosis. Navigation and robot-guided screw placement, disc preparation, osteophyte release, and cage insertion were aided by a retractor-mounted camera. Postoperative imaging showed restored disc height and alignment. Prone LLIF appears feasible and effective, although the evidence remains short-term and limited.

The video can be found here: https://stream.cadmore.media/r10.3171/2026.4.FOCVID2632

## Transcript

In this surgical video, we present the application of a single-position prone LLIF using robotic assistance.

### 0:26 Preoperative Imaging.

This is a case of a 68-year-old female with a history of low back pain as well as right lower extremity pain. After failing a period of conservative treatment, she elected to undergo L3–5 LLIF with posterior fusion.

### 0:39

Standing films show slight coronal deformity with lateral listhesis at L3 and L4. The disc spaces are severely collapsed, causing foraminal stenosis as well.

### 0:50 Positioning/Prone Lateral Bolsters.

The operative plan was to perform a prone lateral, L3–5 360 operation. Here you can see the positioning where side bolsters are placed to allow lateral bend at the approach side. In this case, the right side was selected to approach from the concavity of her coronal curvature. Also note the bolster on the left side acts as a backstop, which is crucial for prone lateral surgery.^1–3^ The incision for the dynamic reference arrays is placed over the right PSIS.

### 1:16

The preoperative CT is merged with interop fluoroscopic images.

### 1:21

Indirect decompression utilizing the LLIF approach was intended for this patient

### 1:30 Pre-CT Planning/Implant Selection.

Here is an overview of the surgical plan. The trajectories for the lateral cages are also visible on the 3D plan and illustrate how one incision can be used for both levels in this case.^2^

### 1:41

In this example, the robotic planning software allows for precise planning of the cage angle and depth as well as planning the trajectory for the incision.

### 1:50 Surgical Procedure.

Screws are placed first after the merge is performed. The rationale here is that the part of the surgery that requires the most accuracy from navigation should be done first prior to manipulation of the spine. This operation can be performed in a lateral decubitus position.^4^ In our experience, for two or more levels, prone lateral helps facilitate screw placement and subsequent run placement as this is more natural for most surgeons.^2,5^

### 2:28

Prior to docking the retractor, in this case there are lateral osteophytes covering the disc space as a result of the coronal degeneration.

Illustrated here is a maneuver using a 10-mm Cobb with navigation guidance to break through these osteophytes.^6^ The hammer is used, followed by a twisting maneuver to mobilize the osteophytes. Since the laterality of the approach is from the concavity of the curvature, osteophytes covering both the L3–4 and L4–5 disc space can be readily addressed through the same opening.

### 2:57

While many of the features of this robotic platform have been previously demonstrated on other navigation platforms, there is somewhat of a nuanced distinction between robotics and navigation. In this example, the planning software is unique to the robotic platform, which allows for real-time adjustment of the planned cage placement. This is especially helpful if there is any axial rotation in the anatomy.

### 3:21

The initial dilator with directional EMG is docked using navigation guidance. Ideally, a start point with a threshold of 10 mA is identified.^2,3^ In the event of a triggered response, the dilator can easily be repositioned. Navigation allows for real-time 3D visualization, and it completely eliminates the need for fluoroscopy while docking. The dilator is secured with a K-wire and is later verified with one fluoroscopic image.

### 3:45

This initial probe has the capability of directionally triggered EMG. In the event of a triggered response below the desired milliamp threshold, several maneuvers can be undertaken with navigation guidance. First, the dilator can be placed more anteriorly through the psoas and swept dorsally to sweep the lumbar plexus away from the retractor docking point. Another maneuver is the table tilt, where the table is tilted away and the dilator is docked more anteriorly at a slightly oblique angle. Once a safe docking point is found, the retractor can then be tilted more orthogonal to the disc space.

### 4:27 Disc Preparation.

While starting the disc prep, one can visualize the opening made by the Cobb during the initial maneuver to break the osteophytes. The disc space is then prepped in a fairly standard fashion with most transpsoas lateral techniques.^6^ The Cobb is advanced across the disc space to break the contralateral annulus. Again, navigation helps facilitate orientation of the instrument within 3 dimensions.

### 4:46

The video here is from a retractor-mounted 4K camera that we use specifically for prone lateral cases. In our technique, this allows for enhanced ergonomics by allowing the surgeon to work with their arms in a relaxed position without having to directly visualize through the retractor. This, coupled with the navigation guidance, allows for, in our experience, an optimized workflow for prone lateral approaches. Fluoroscopy can be utilized at any point to verify the accuracy of the navigation. In our usual workflow, we typically will obtain a fluoroscopic image after placement of the wire, and after placement of the 5-mm-height trial. In some cases, particularly when the disc space is collapsed, we will also image the 10-mm Cobb elevator to ensure that the instrument is within the disc space.

### 5:39 Placement of Trial.

This is followed by a disc trial up to the desired height.

### 5:55 Placement of Cage.

Finally, a cage is placed again using navigation as guidance. In this case, expandable cages are used, although the technique is applicable to monolithic cages as well.^2,6^

### 6:15

The combination of navigation and the retractor-mounted camera helps facilitate surgical training, and a preceptor could readily observe a trainee performing this case in a safe fashion.^5^

### 6:25

On average, a two-level prone LLIF 360 takes 2 hours and 34 minutes from start to finish. The efficiency that can be gained in this approach’s rod placement can be done while the lateral incision is closed. Theoretically, both the initial screw placement and the lateral exposure can be done simultaneously by two surgeons for even greater efficiency. For training purposes, our practice is to perform these steps separately, however.

### 6:50 Postoperative Imaging/X-Ray.

Postoperative images show improvement of coronal alignment as well as restoration of disc height at the operative levels.^1,2^ This patient did well and was discharged home on postoperative day 2.

## Acknowledgments

We thank Katie H. Hurrish, PhD, for editorial assistance.

## Disclosures

V. Chang reported personal fees from Globus Medical, Viseon, Bioventus, and SI-BONE; and grants from BCBSM outside the submitted work.

## Author Contributions

Primary surgeon: Chang. Assistant surgeon: Yeo, Bartlett, Reese. Editing and drafting the video and abstract: all authors. Critically revising the work: Chang, Al Tekreeti, Reese, Chaker. Reviewed submitted version of the work: Chang, Reese, Chaker. Approved the final version of the work on behalf of all authors: Chang. Supervision: Chang.

## Supplemental Information

### Patient Informed Consent

The necessary patient informed consent was obtained in this study.
